# Measurement of ultrasonic phase and group velocities in human dental hard tissue

**DOI:** 10.1186/2050-5736-1-5

**Published:** 2013-05-01

**Authors:** Sleiman R Ghorayeb, Panagiotis Petrakis, Michael McGrath, Ben A Scheven

**Affiliations:** 1 School of Engineering and Applied Sciences, Ultrasound Research Laboratory, Hofstra University, 104 Weed Hall, Hempstead, NY 11549, USA; 2 Orthopaedics Research Lab - FIMR, North Shore Hospital, Manhassett NY, USA; 3 School of Dentistry, College of Medical and Dental Sciences, University of Birmingham, Birmingham, UK

**Keywords:** Dental ultrasound, Therapeutic ultrasound, Tooth repair, Phase velocities, Slowness curves

## Abstract

**Background:**

The development of ultrasound for use in dental tissues is hampered by the complex, multilayered nature of the teeth. The purpose of this preliminary study was to obtain the phase and group velocities associated with several directions of ultrasonic wave propagation in relation to the tooth structure, which would then lead to the determination of the elastic constants in dental hard tissue. Knowledge of these elastic constants can be used to feed back into numerical models (such as finite element) in order to simulate/predict ultrasonic wave propagation and behavior in the teeth. This will help to optimize ultrasonic protocols as potential noninvasive therapeutic tools for novel dental regenerative therapies.

**Methods:**

An extracted human second molar was used to determine time-of-flight information from A-scan signatures obtained at various angles of inclination and rotation using a scanning acoustic microscope at 10 MHz. Phase and group velocities and associated slowness curves were calculated in order to determine the independent elastic constants in the human teeth.

**Results:**

Results show that as the tooth was inclined at three azimuthal angles (Θ_in_ = 0°, 15°, and 30°) and rotated from Φ_in_ = 0° to 360° in order to cover the whole perimeter of the tooth, slowness curves constructed from the computed phase and group velocities versus angle of rotation confirm the inhomogeneous and anisotropic nature of the tooth as indicated by the nonuniform appearance of uneven circular shape patterns of the measurements when compared to those produced in a control isotropic fused quartz sample.

**Conclusions:**

This study demonstrates that phase and group velocities of ultrasound as determined by acoustic microscopy change and are dependent on the direction of the tooth structure. Thus, these results confirm that the tooth is indeed a multilayered anisotropic structure underscoring that there is no single elastic constant sufficient to represent the complex structure of the tooth. The findings underline the importance to take into account these crucial characteristics in order to develop and optimize therapeutic as well as diagnostic applications of ultrasound in dental tissue repair, and further studies are warranted to analyze ultrasound transmission at various frequencies and intensities in different teeth to develop appropriate models for ultrasound biophysical behavior in dental tissues.

## Background

Ultrasonic waves such as those used routinely during medical examinations and prognoses propagate in a very complex manner. This level of complexity holds true even when these sound waves propagate in ideal, homogeneous, and uniform media. Despite the fact that diagnostic ultrasound techniques are based on well-established phenomena, there remains the area of dentistry where ultrasound has not played yet a very important role. This is due to the fact that ultrasonic propagation in inhomogeneous/anisotropic media results in further degradations in acoustic field. Scattering effects at the microscale level as well as at the multilayer interfaces are responsible for these degradations.

In earlier diagnostic ultrasound experiments performed on the human teeth, an ultrasonic pulse-echo system was able to detect the enamel-dentin junction and the dentin-pulp interface [1]. Ultrasonic imaging techniques have also been applied to other areas of dentistry, specifically prosthodontics. A B-mode ultrasonic system for determining the thickness of the masticatory mucosa for denture construction has also been developed [2], and the use of the scanning acoustic microscope to image the elastic properties of carious human dental enamel has been described [3]. Acoustic microscopy has been successful for imaging lesions in tooth enamel and detecting the enamel-dentin interface [4]. Finite element models [5,6] as well as PSpice (OrCAD™, Cadence Design Systems, Inc., San Jose, CA, USA) transmission line (*T*-line) models [7] used to understand ultrasonic wave propagation in the teeth have been established. In order to confirm predictions made by these latter finite element and *T*-line models, experimental data was gathered [8] for the purpose of assessing the viability of ultrasound to detect cavities and fractures in the extracted human teeth. The method showed great promise as a potential improvement upon current dental imaging systems.

For years, the main applications of therapeutic ultrasound in dentistry have been mostly limited to oral surface cleaning (i.e., removal of plaque and calculus) or root canal treatment [9]. In recent years, however, therapeutic ultrasound studies performed on dental tissue have taken a different path [10,11]. The goal of this research is to develop novel ultrasound therapies that can be easily applied to the teeth to promote tissue healing, more specifically tooth regeneration. Preliminary results showed that this idea of repairing the teeth with ultrasound has potential. The tooth is a hard mineralized structure with a living soft tissue core capable to respond to outside stimuli including physical forces. Research has demonstrated that ultrasound in the low frequency range, generally used in dentistry for dental scaling, stimulated gene expression and the production of tissue regulatory growth factors that are considered to be crucial for odontoblast differentiation, activity, and dentin repair [12,13]. More recently, it was demonstrated that low-frequency ultrasound stimulated differentiation and *in vitro* mineralization in odontoblast-like cultures [11]. These findings are novel and exciting and support the working hypothesis that ultrasound may be an effective noninvasive tool for novel dental regenerative therapies [14]. Interestingly, these findings were recently corroborated in *in vitro* tooth slice organ cultures where low-intensity pulsed ultrasound was shown to increase pre-dentin thickness and sub-odontoblast cell numbers [10,15].

However, to develop ultrasound as a therapeutic application, a thorough understanding is required regarding ultrasonic wave propagation and intensity distribution in teeth structures. The tooth is a highly anisotropic medium. This quality stems from two different criteria—layer consistency and structural component. The enamel portion of a tooth mainly consists of calcium hydroxyapatite [16]; the dentin represents a collagenous mineralized substance much like the bone and contains tubular canals, and the tooth's central cavity (pulp) contains a loose connective tissue with various cell types, blood vessels, and nerves. Odontoblasts surround the dental pulp with cellular processes extending into the dentinal tubules which may also contain neurite endings. In addition, the root is enclosed by a thin layer of bone-like material called cementum, which is surrounded by a periodontal ligament. The latter tissue is highly cellular and contains bundles of thick collagenous fibers responsible for attaching the tooth to the alveolar jaw bone. So, as it may be appreciated, the tooth is composed of a very complex mesh of intricate layers, each having different chemical and structural characteristics than the one adjacent to it. As a result of all these effects, ultrasonic waves propagating within a tooth would undergo a fair amount of mode conversion. This, in a way, is similar to the assessment of the structural integrity of anisotropic components used in aircrafts and submarines whereby elastic constants of the host material need to be determined.

In this paper, we propose and test a proof-of-concept methodology based on previous studies [17-21] that would eventually lead to the determination of the elastic constants in dental hard tissue. The study used an extracted human tooth to determine phase and group velocities from measured time-of-flight (TOF) of an ultrasonic wave as it propagates along arbitrary directions through the tooth crown of the specimen using a pulse-echo setup at different inclination angles of incidence.

## Methods

All studies were approved by and in compliance with the guidelines set by the institutional review board committee at Hofstra University (Hempstead, NY, USA).

### Anisotropy consideration

Assuming a tooth can be classified as a simple orthorhombic material, nine independent elastic constants need to be determined. These are represented by the matrix in Equation 1:

(1) $$C_{\mathrm{ij}}=\left[\begin{array}{cccccc} C_{11} & C_{12} & C_{13} & 0 & 0 & 0\\ & C_{22} & C_{23} & 0 & 0 & 0\\ & & C_{33} & 0 & 0 & 0\\ & & & C_{44} & 0 & 0\\ & \mathrm{SYM}. & & & C_{55} & 0\\ & & & & & C_{66} \end{array}\right].$$

The elastic constants along the diagonal are defined in the <1 0 0>, <0 1 0>, and <0 0 1> directions. In other words, the waves that are traveling along these directions are considered to be pure mode, and their velocities (i.e., longitudinal (*V*_L_), shear horizontal (*V*_SH_), and shear vertical (*V*_SV_)) are determined along these directions. Note that all diagonal elastic constants can be determined by propagation waves in symmetric directions. The waves traveling along the lower-symmetry directions are not pure modes. The behavior of elastic waves propagating in anisotropic materials [17] is governed by the following:

(2) $$C_{\mathrm{ijkl}}\left(\frac{\partial^{2}u_{l}}{\partial x_{j}\partial x_{k}}\right)=\rho {\ddot{u}}_{i},$$

where *C*_*ijkl*_ is the elastic constant, *u*_*i*_ is the displacement, and ρ is the mass density of the material. It is assumed that a plane wave solution *u*_*i*_, with wave vector *k*, frequency ω, and amplitude *A*_*i*_, of the form *u*_*i*_ = *A*_*i*_*e*^*i*(*kx −* ω*t*)^ exists and can be substituted back into the equation of motion (Equation 2) in order to obtain Kelvin-Christoffel elastic stiffness equation as follows:

(3) $$\left(\Gamma_{\mathrm{ik}}-\rho \;V^{2}\delta_{\mathrm{ik}}\right)u_{k}=0,$$

where *Γ*_*ik*_ = *C*_*ijkl*_*α*_*i*_*α*_*j*_ are Christoffel elastic stiffnesses [17], *V* is the phase velocity, and α_*i*_ and α_*j*_ are the direction cosines of propagating wave. A general solution to (Equation 3) gives relationships between the phase velocities *V* and *C*_*ij*_ for the wave propagation along arbitrary directions. As an example, in the <0 1 1> direction, we should have the following:

(4) $$\frac{1}{2}\left[\begin{array}{ccc} C_{55}+C_{66} & 0 & 0\\ 0 & C_{22}+C_{44} & C_{23}+C_{44}\\ 0 & C_{23}+C_{44} & C_{33}+C_{44} \end{array}\right],$$

then, using the solution of Kelvin-Christoffel's equation, we get the following:

$$\rho \;V^{2}=\frac{1}{2}\left(C_{55}+C_{66}\right)$$

or

(5) $$\rho V^{2}=\frac{1}{2}\left\{C_{22}+C_{33}\pm {\left[{\left(C_{22}–C_{33}\right)}^{2}+4\;{\left(C_{23}+C_{44}\right)}^{2}\right]}^{\frac{1}{2}}\right\}\;+C_{44}.$$

If *C*_*ii*_ is known and ρ*V*^2^ is measured, then *C*_23_ can be calculated as follows:

(6) $$C_{23}=\pm {\left\{{\left[2\rho V^{2}–\frac{1}{2}\left(C_{22}+C_{23}+2C_{44}\right)\right]}^{2}–\frac{1}{4}{\left(C_{33}-C_{22}\right)}^{2}\right\}}^{\frac{1}{2}}-C_{44}.$$

Similarly, using the velocities along the <1 0 1> and the <1 1 0> directions, one can obtain *C*_13_ and *C*_12_ as follows:

(7) $$C_{13}=\pm {\left\{{\left[2\rho V^{2}–\frac{1}{2}\left(C_{11}+C_{33}+2C_{55}\right)\right]}^{2}–\frac{1}{4}{\left(C_{33}–C_{11}\right)}^{2}\right\}}^{\frac{1}{2}}-C_{55},$$

(8) $$C_{12}=\pm {\left\{{\left[2\rho V^{2}–\frac{1}{2}\left(C_{11}+C_{33}+2C_{66}\right)\right]}^{2}–\frac{1}{4}{\left(C_{22}–C_{11}\right)}^{2}\right\}}^{\frac{1}{2}}-C_{66}.$$

The above description says that one can measure the wave velocities along the <1 0 0>, <0 1 0>, and <0 0 1> directions for the diagonal elements *C*_11_, *C*_22_, *C*_33_, *C*_44_, *C*_55_, and *C*_66_ and measure the wave velocities along the <0 1 1>, <1 0 1>, and <1 1 0> directions for the off-diagonal elements *C*_23_, *C*_13_, and *C*_12_. One should appreciate that this is not a trivial procedure. In order to measure the velocities along the aforementioned directions, four similar (if not exact) teeth specimens need to be prepared as follows:

1. All surfaces are perpendicular to the symmetry direction in such a way that the length in each direction is at least five longitudinal wavelengths for proper measurement, and all surfaces are flat.

2. Largest surface perpendicular to the <0 1 1> direction and upper and lower surfaces are flat.

3. Largest surface perpendicular to the <1 0 1> direction and upper and lower surfaces are flat.

4. Largest surface perpendicular to the <1 1 0> direction and upper and lower surfaces are flat.

However, as mentioned earlier, this study is only a proof-of-concept and was performed on only one tooth sample. Future studies will consider the aforementioned implementation to measure the elastic constants from obtained phase velocities.

### Experimental setup

**Figure 1:**
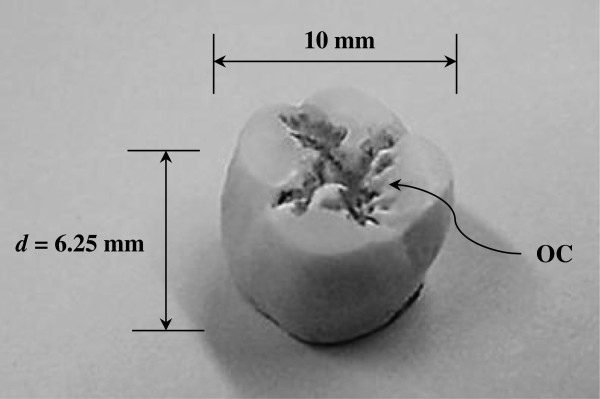
Flattened adult human second molar specimen used for study showing the natural occlusions (OC).

It should be emphasized that teeth come in a variety of shapes and sizes (different morphologies—e.g., incisors and molars). Also, the teeth are so brittle that extreme care must be taken during the cutting process so that they do not get fragmented. In this study, an extracted adult human (age, sex, or race not known) second molar was tested using a phosphate buffered saline (PBS) solution as the coupling medium. The tooth was sterilized with an autoclave system and was completely dry with no vital root. In fact, the latter was not needed in this study since the focus was on the determination of material properties associated with specifically the dentin layer as described below. Since the study deals with the determination of physical tooth characteristics (wave velocities and eventually elastic constants), both the top surface of the crown and the bottom cusp surface surrounding the pulp chamber were flattened by grinding to maintain good wave propagation that was not hindered by uneven surfaces. This resulted in a miniaturized tooth size of *d* = 6.25 mm in length (as opposed to the 15-mm original length) and 10 mm in width (measured across the breadth of the crown area) as shown in Figure 1. However, some of the indentations due to natural occlusions (OC in Figure 1) were left. Also, one should keep in mind that due to the flattening process, the majority of the enamel layer has been lost, exposing only the dentin portion of the tooth. This in fact is advantageous since the major anisotropy/inhomogeneity is present in the dentin due to the presence of collagenous mineralized substance and tubular canals, as mentioned earlier.

**Figure 2:**
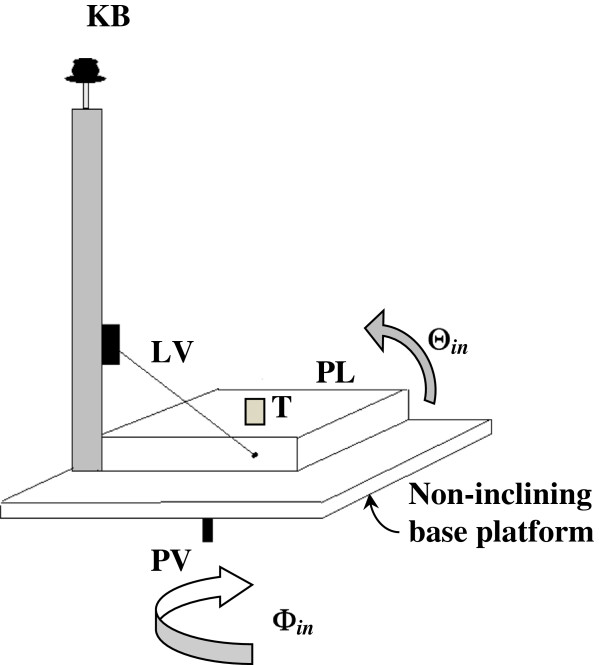
**Plexiglas tooth holder showing the inclining *Θ***_**in **_**platform (*PL*) that holds the tooth (*T*). ***Θ*_in_ is controlled by a turning knob (*KB*) that controls a lever (*LV*) connected to (*PL*). A base pivot (*PV*) controls the angle of rotation *Φ*_in_.

**Figure 3:**
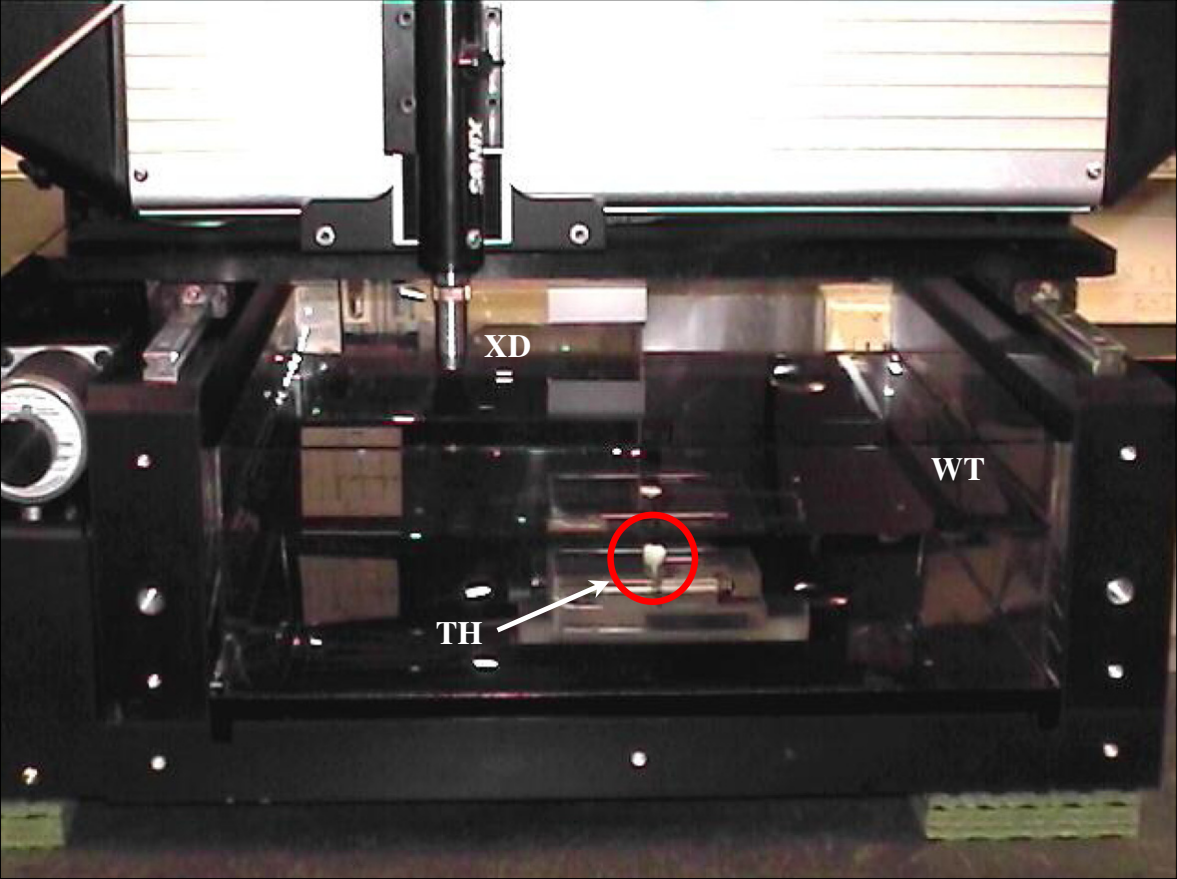
**Experimental setup in the water tank.** It shows the relative position of the ultrasound transducer (*XD*) and the tooth sample (*TH*; shown in *red circle*) in the water tank (*WT*).

The A-scan signals were acquired using a scanning acoustic microscope consisting of an ultrasonic pulser/receiver (Model 5800PR, Olympus-Panametrics, Waltham, MA, USA), an immersion transducer (Model V312, 10 MHz, 1.27 cm (0.5 in.) focal length, 0.635 cm (0.25 in.) aperture diameter, Olympus-Panametrics, Waltham, MA, USA), a FlexSCAN-C® ultrasonic C-scan system (Sonix Inc., Springfield, VA, USA) with a tank containing PBS solution as the couplant, and a computer software system with a front-wall-follower gating capability. A Plexiglas™ tooth holder (Figure 2) was machined to hold the tooth under test in an upright position with the top of the crown facing the radiated acoustic field. A special rotating and inclining base was constructed in order for the ultrasonic beams to propagate at various angles in the tooth. Since the transducer could not rotate around the tooth or its axis, the device was made to rotate 360° around a pivot in the tank and enabled the determination of the TOF values at different angles (rotational *Φ*_in_ and azimuthal/inclination *Θ*_in_). During data acquisition, it is important to note that the tooth was scanned with the transducer perpendicular to the tooth surface of the crown as shown in Figure 3.

**Figure 4:**
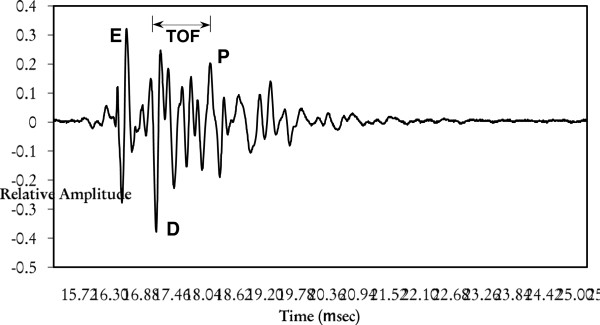
**A typical received ultrasound A-scan signal from a normal tooth and corresponding layers.** (*E*, enamel; *D*, dentin; *P*, pulp) It shows how the TOF is determined.

The transducer is designed for negative spike excitation. The maximum spike excitation voltage is 300 V with a fast rise time and short duration. Although negative spike excitation is recommended, continuous wave or tone burst excitations may be used. It is helpful to note that the number of cycles per tone burst excitation depends on the duty cycle, which itself depends on several other factors such as impedance, total power, voltage, and phase of the transducer. The spatial length of the pulse for the transducer used in this experiment has been determined to be about 0.2 μs, which is small compared to the echoes obtained from the tooth layers (enamel, dentin, tubular canals, and pulp). The pulser/receiver was set to send an ultrasonic pulse with an energy setting between 50 and 100 μJ and a gain between 20 and 40 dB to insure proper propagation in the tooth sample. The tooth (mounted in the fixture) was placed in the water tank in the focal plane within the transducer's cone of insonification. The FlexSCAN-C® system was used to examine the front-wall reflection off the crown and all subsurface layers so that the TOF can be adequately measured for speed determination. It is crucial to point out that the resulting A-scan is composed of multiple modes (longitudinal, edge longitudinal, edge shear, head, and surface) of the ultrasound wave transmitted as a result of mode conversion [8]. As an example, Figure 4 shows a typical RF A-mode scan (amplitude vs. time) from a normal unflattened tooth sample similar to the one we are investigating. The boundary of each of the three major layers (enamel, dentin, and pulp) is clearly indicated in this figure. However, in our case, since the enamel layer has been removed due to the filing/flattening of the tooth, only the peaks corresponding to the dentin and the pulp were used for calculating the TOF.

As noted earlier, the propagation of ultrasonic energy impinging on the anisotropic sample at arbitrary (orthogonal or oblique) angles results in three mode-converted waves. These can be observed by measuring the TOF along the energy path. The phase velocities needed for the determination of the elastic constants are a function of the propagation time. For an arbitrary angle of incidence (*Φ*_in_ and *Θ*_in_), the phase velocity [17] is given by the following:

(9) $$V_{p\left(\Theta_{\mathrm{in}},\Phi_{\mathrm{in}}\right)}={\left[{\left(\frac{\mathrm{\Delta t}\left(\Theta_{\mathrm{in}},\Phi_{\mathrm{in}}\right)}{d}\right)}^{2}-2\frac{\mathrm{\Delta t}\left(\Theta_{\mathrm{in}},\Phi_{\mathrm{in}}\right)\cos\Theta_{\mathrm{in}}}{V_{w}d}+{\left(\frac{1}{V_{w}}\right)}^{2}\right]}^{-\frac{1}{2}},$$

where Δ*t* is the one-way TOF, *d* is the thickness of the tooth (as shown in Figure 1), and *V*_*w*_ is the phase velocity in the PBS solution. Furthermore, using Snell's law, the angle of refraction *Θ*_*r*_ can be determined from the following:

(10) $$\cos\Theta_{r}=\sqrt{\left(1-{\left(\frac{V_{w}}{V_{p}\left(\Theta_{\mathrm{in}},\Phi_{\mathrm{in}}\right)}\right)}^{2}\right)}.$$

From Equations 9 and 10, the magnitude and directions of phase velocities for all modes of propagation inside the tooth can be determined for any arbitrary angle of incidence, *Θ*_in_. Once this is done, an inversion routine based on the Newton-Rhapson method [17] can be implemented on the above characteristic polynomial to determine the elastic constants. Knowledge of these elastic constants can be used to feed back into models (such as finite element) in order to simulate/predict ultrasonic wave propagation and behavior in the teeth.

Furthermore, once the angle of refraction *Θ*_*r*_ has been calculated for each phase velocity, one can determine the various group velocities *V*_*g*_ which correspond to these respective phase velocities at the calculated angles *Θ*_*r*_. These group velocities represent the actual family of longitudinal stress-wave velocities in those particular directions within the tooth. Group velocities can be determined by projecting their vectors, on the surface of the slowness curves (as will be shown later), normal to the corresponding phase velocities. Using simple trigonometry, the group velocities can then be computed from the following:

(11) $$V_{g}=\frac{V_{p}}{\cos\Theta_{r}}.$$

## Results and discussion

**Figure 5:**
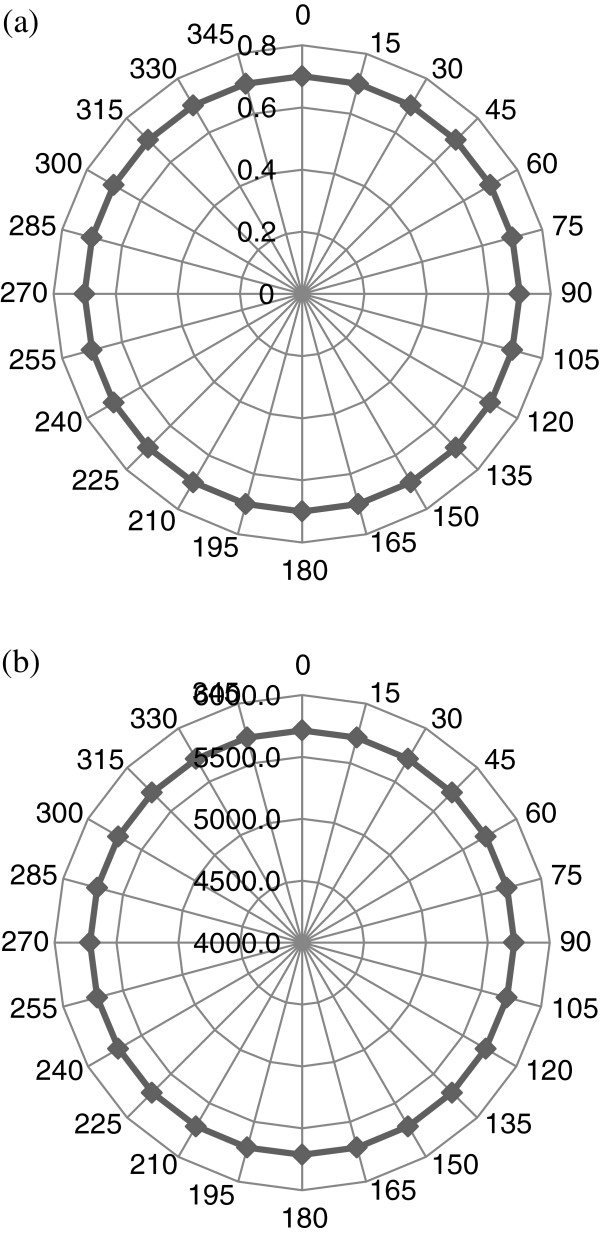
**Panoramic polar plots of (a) one-way TOF (μs) and (b) measured velocities (m/s) for quartz sample.** The plots were generated at an angle of inclination *Θ*_in_ = 0°.

**Figure 6:**
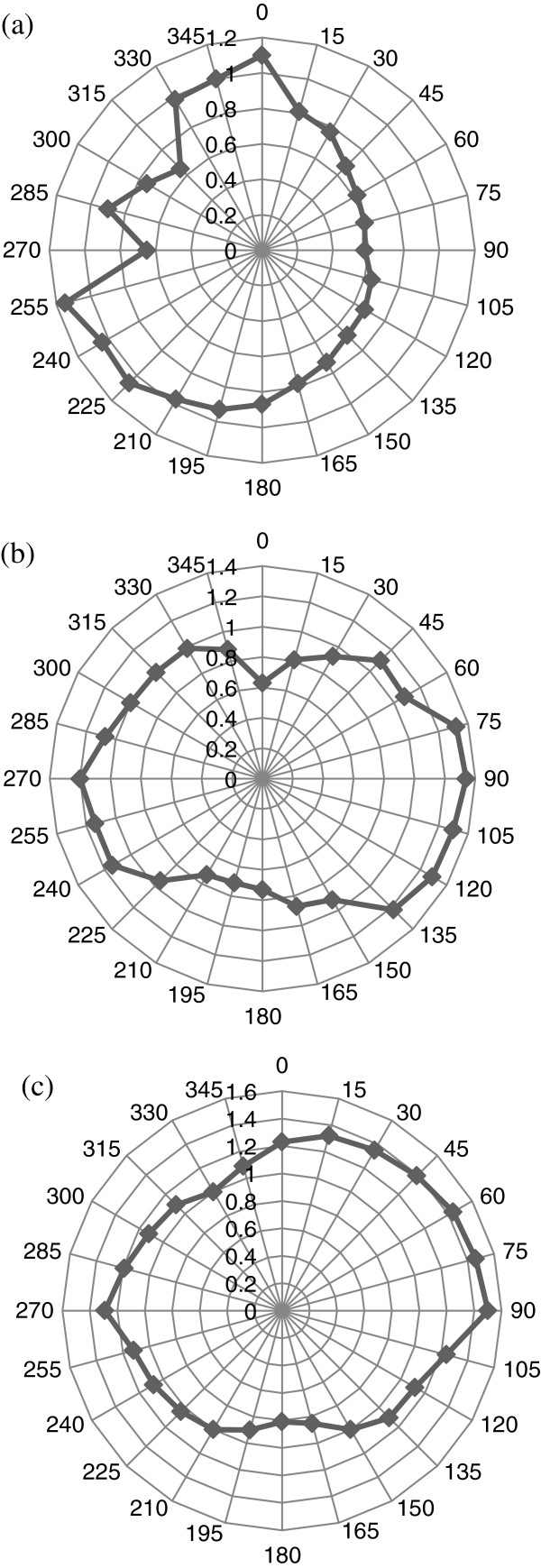
**Panoramic polar plot of the one way TOF (μs) in tooth for all three angles of inclination.** (**a**) *Θ*_in_ = 0°, (**b**) *Θ*_in_ = 15°, and (**c**) *Θ*_in_ = 30°.

**Figure 7:**
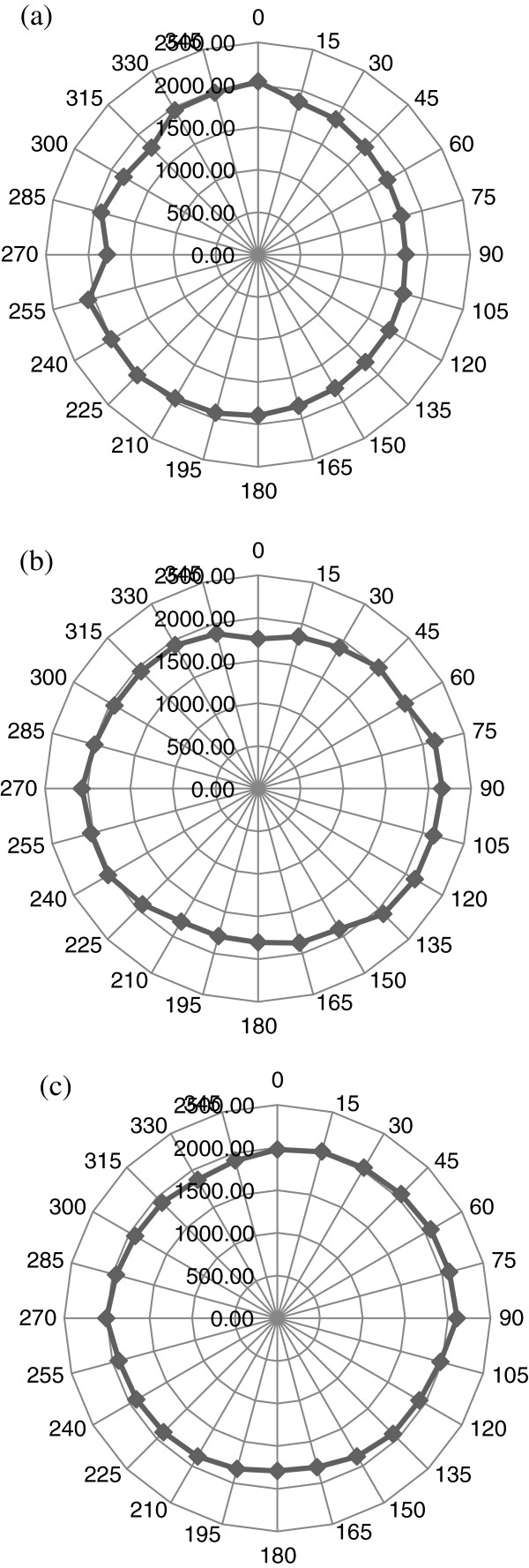
**Corresponding panoramic polar plots of phase velocities (m/s) in tooth for all three angles of inclination.** (**a**) *Θ*_in_ = 0°, (**b**) *Θ*_in_ = 15°, and (**c**) *Θ*_in_ = 30°.

Several tests were performed for obtaining TOF information using three azimuthal angles (angles of inclination) set at *Θ*_in_ = 0°, 15°, and 30°. For each of these three angles, the platform was rotated from *Φ*_in_ = 0° to about 360° in order to cover the whole perimeter of the tooth. The phase velocity in the PBS solution (*V*_*w*_) is assumed to be similar to that in water (1,500 m/s). For each set of incident angle (*Φ*_in_, *Θ*_in_), TOF and the phase velocity *V*_*p*_ were determined. Polar plots of TOF and slowness curves from the computed phase velocities are then constructed. In order to confirm the experimental measurement system, TOF measurements as a function of *Φ*_in_ and *Θ*_in_ were made on an isotropic, homogeneous square piece of fused quartz plate of 4-mm thickness. The polar plot of TOF values Δ*t* ((*Φ*_in_, 0° → 2*π*), *Θ*_in_) determined from around the quartz plate is perfectly circular for propagation of longitudinal wave, with a constant longitudinal velocity of about 5,714 m/s. This observation confirms the homogenous characteristic of the control quartz plate. Figure 5 shows the results for both TOF and longitudinal velocities obtained for the quartz sample at an angle of inclination (*Θ*_in_ = 0°). Figure 6 shows the TOF measurements for the tooth for all three angles of inclination (*Θ*_in_ = 0°, 15°, and 30°), and Figure 7 illustrates the corresponding phase velocities in the tooth for the same angles.

**Figure 8:**
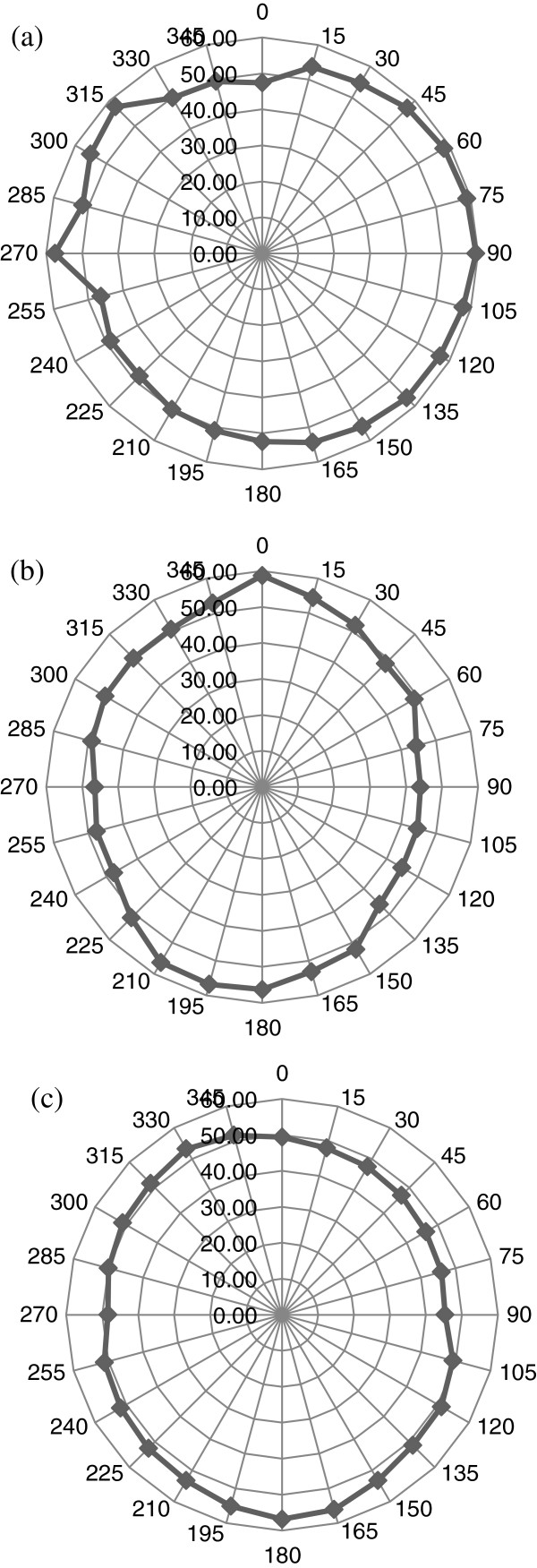
**Panoramic polar plots of direction cosines (degrees) of phase velocities in tooth for all three angles of inclination.** (**a**) *Θ*_in_ = 0°, (**b**) *Θ*_in_ = 15°, and (**c**) *Θ*_in_ = 30°.

The direction cosines of the phase vectors in the tooth can be determined from Equation 10. These are shown in Figure 8.

The observations highlight polar/slowness curves (Figures 6 and 7) which confirm the inhomogeneous and anisotropic nature of the tooth. This is indicated by the nonuniform appearance of uneven circular shape patterns of both TOF and *V*_*p*_ measurements when compared with the perfect circular measurements made in the control isotropic fused quartz sample shown in Figure 5. This outcome has obvious implications for the development of ultrasound as a diagnostic tool, and in this respect, further analyses need to be conducted to determine the phase velocity of ultrasound at different frequencies.

**Figure 9:**
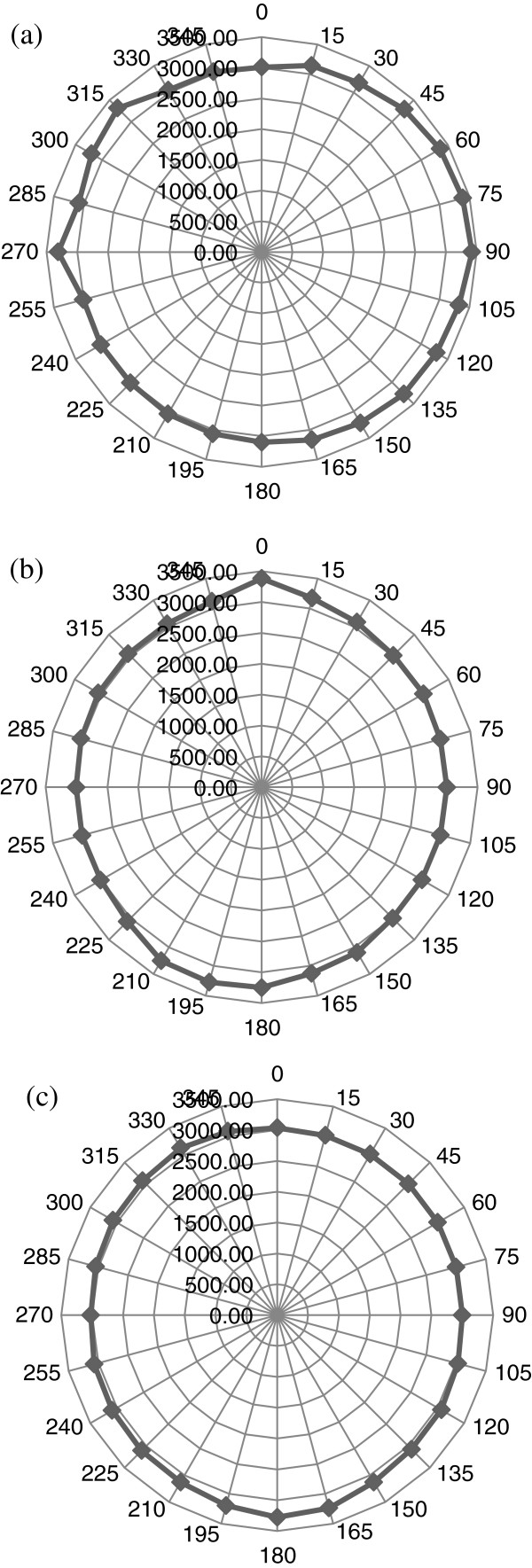
**Corresponding panoramic polar plots of group velocities (m/s) in tooth for all three angles of inclination.** (**a**) *Θ*_in_ = 0°, (**b**) *Θ*_in_ = 15°, and (**c**) *Θ*_in_ = 30°.

The results as shown in Figures 7 and 8 will help in the complete determination of the magnitude and directions of phase velocities for all three modes of wave propagation (quasi-longitudinal and two quasi-shear) inside the tooth for any arbitrary angle of incidence. These will then be used to determine the independent elastic constants of this orthotropic medium. Also, using Equation 11 in conjunction with the TOF and *V*_*p*_ measurements shown in Figures 7 and 8, the group velocities can be calculated. Figure 9 shows the corresponding polar plots.

As mentioned earlier, since the flattening process of the tooth caused the majority of the enamel layer to be lost, exposing only the dentin portion, the average group velocities attained were approximately 3,178, 3,088, and 3,080 m/s for each of the three azimuthal angles (*Θ*_in_ = 0°, 15°, and 30°), respectively. These values confirm what was reported in earlier studies [1,5-8].

Moreover, regarding the potential use of ultrasound for dental tissue repair, it is important to relate the physical behavior of ultrasound through the different layers of the teeth to those in a viable pulp cavity and relate these characteristics to localized ultrasound intensity in order to assess the biological effects of ultrasound in the pulp region. In other words, knowledge of the elastic constants can be used to feed back into numerical models (such as finite element) in order to simulate/predict ultrasonic wave propagation and behavior in the teeth. This will help optimize ultrasonic protocols as potential noninvasive tools for novel dental regenerative therapies.

## Conclusions

This proof-of-concept study demonstrated that phase velocities (and corresponding group velocities) of ultrasound as determined by acoustic microscopy change and are dependent on the direction of the tooth structure. The data underscore the anisotropic structure and demonstrate that no single elastic constant will be sufficient to represent the complex nature of the tooth. The findings have obvious implications for the development of diagnostic and therapeutic ultrasonic devices for dental tissues, and further correlation studies that will examine other samples are warranted to analyze ultrasound transmission at various frequencies and intensities in different teeth to develop appropriate models for ultrasound biophysical behavior in dental tissues.

## Competing interests

The authors declare that they have no competing interests.

## Authors’ contributions

SRG mentored this research project, provided guidelines and protocols for all measurements and *in vitro* studies, analysis, and interpretation of the data, and wrote the final manuscript. PP and MM carried out the laboratory measurements and interpretation of the data, and participated in the initial drafting of the manuscript. BAS provided clinical information about this application and participated in the interpretation of the data and the initial review of the manuscript. All authors read and approved the final manuscript.
